# Socioeconomic inequalities of outpatient and inpatient service utilization in China: personal and regional perspectives

**DOI:** 10.1186/s12939-017-0706-8

**Published:** 2017-12-04

**Authors:** Dawei Zhu, Na Guo, Jian Wang, Stephen Nicholas, Li Chen

**Affiliations:** 1 Center for Health Policy and Management, Institute of Medical Information & Library, Chinese Academy of Medical Sciences & Peking Union Medical College, Beijing, 100020 China; 2 China Population and Development Research Center, Beijing, 100081 China; 3 School of Public Health, Shandong University, Jinan, 265400 China; 4 School of Management and School of Economics, Tianjin Normal University, Tianjin, 300074 China; 5 Guangdong Research Institute of International Strategies, Guangdong University of Foreign Studies, Guangzhou, 510420 China; 6 Beijing Foreign Studies University, Beijing, 100089 China; 7 Newcastle Business School, University of Newcastle, Newcastle, 2308 NSW Australia; 8 Georgia Prevention Institute, Department of Population Health Sciences, Medical College of Georgia, Augusta University, Augusta, 30912 GA USA

**Keywords:** Health care utilization, Inequality, Resource allocation, Health policy

## Abstract

**Background:**

China’s health system has shown remarkable progress in health provision and health outcomes in recent decades, however inequality in health care utilization persists and poses a serious social problem. While government pro-poor health policies addressed affordability as the major obstacle to equality in health care access, this policy direction deserves further examination. Our study examines the issue of health care inequalities in China, analyzing both regional and individual socioeconomic factors associated with the inequality, and provides evidence to improve governmental health policies.

**Methods:**

The China Health and Nutrition Survey (CHNS) 1991–2011 data were used to analyze the inequality of health care utilization. The random effects logistic regression technique was used to model health care utilization as the dependent variable, and income and regional location as the independent variables, controlling for individuals’ age, gender, marital status, education, health insurance, body mass index (BMI), and period variations. The dynamic trend of 1991–2011 regional disparities was estimated using an interaction term between the regional group dummy and the wave dummy.

**Results:**

The probability of using outpatient service and inpatient services during the previous 4 weeks was 8.6 and 1.1% respectively. Compared to urban residents, suburban (OR: 0.802, 95% CI: 0.720–0.893), town (OR: 0.722, 95% CI: 0.648–0.804), rich (OR: 0.728, 95% CI: 0.656–0.807) and poor village (OR: 0.778, 95% CI: 0.698–0.868) residents were less likely to use outpatient service; and rich (OR: 0.609, 95% CI: 0.472–0.785) and poor village (OR: 0.752, 95% CI: 0. 576–0.983) residents were less likely to use inpatient health care. But the differences between income groups were not significant, except the differences between top and bottom income group in outpatient service use.

**Conclusion:**

Regional location was a more important factor than individual characteristics in determining access to health care. Besides demand-side subsidies, Chinese policy makers should pay enhanced attention to health care resource allocation to address inequity in health care access.

## Background

Since the 1978 reform and opening up period, China has experienced enormous demographic and socio-economic changes. China’s gross domestic product (GDP) per capita increased from $US1145 in 2000 to $US8016 in 2015 [1], and China has also made remarkable progress in the development of its health care system. For example, practicing (assistant) physicians per thousand population grew from 1.68 in 2000 to 2.12 in 2014 and the life expectancy of the Chinese population increased by 4 years [2]. However, rapid growth and longevity has brought increased income inequality, with the individual income Gini coefficient rising from 0.401 in 2000 to 0.462 in 2015. Mirroring China’s income inequalities, the gap between the rich and the poor in access to health care has also widened. There is strong evidence of pro-rich inequality in China health system [3, 4]. By 2014, the average yearly health care expenditure was $US189.05 among urban residents, but only $US109.22 among rural residents. Of course, inequality of health care utilization is not unique to China, but also exits in many other developing [5, 6] and developed countries [7–10]. Studies conducted in a number of developed and developing countries also found that rich residents have a higher probability of obtaining health care when sick than the poor. Therefore, improving health care equity and closing the gap between the rich and poor in accessing health care have become priorities for health systems in many countries and organizations [11–13].

Equal access to qualified health care has two major components, affordability and availability [14]. Affordability and availability of health care services are two sides of the same coin when seen from the individual and regional perspectives. Affordability of health care, mainly related to household income, health insurance reimbursement rates and other income-related factors, has received the most attention in assessing health systems and improving their performance. The availability of health care describes access from the regional level, which is linked mainly to health care resource allocations, governmental funding and government policies.

Different causes of inequity should be tackled with different corresponding compensation strategies. Enhancing the affordability mostly refers to demand-side financing strategies such as pro-poor subsidies and insurance for low-income residents schemes [15]. But regional factors that compromise the availability of health care services should be corrected with supply-side compensation, like grants for health care infrastructure and salary subsidies for health workers. In recent years, demand-side subsidies have been extensively applied to address health care access. Researchers and policy makers argue that demand-side financing is not only better at targeting subsidies to the poor, but by linking subsidies with output, they also provide the right incentives for efficiency [16, 17]. Supply-side financing strategies have been criticized for their inefficiencies [16, 18].

Recent research has resulted in some unexpected findings that are incompatible with the above supply-side versus demand-side intuitions concerning inequalities. There is no unanimity in the research on health care equality that shows that affordability is a more important cause of (in)equality of service utilization than availability. Feng et al. found that regional factors were more significant indicators of hospital births in China than individual income [19] and Li et al. showed that urban–rural and core-periphery gaps were significant determinants in health care access in Henan province [20]. Van Doorslaer et al. found no evidence of income-related inequity in GP visits in European countries [8] and M.Makinen et al. found that in developing countries, the richer households did not devote a consistently higher percentage of their consumption expenditures to health care [5].

Without careful examination, demand and supply-side intuitions concerning health care inequalities can result in misguided policy-making. Health care financing strategies employed in China and other countries require careful analysis. This study analyzes the personal and regional socioeconomic factors associated with inequalities in health care utilization in China and provides evidence and recommendations for improving governmental health policy financing.

## Methods

### Data sources

Data were obtained from the China Health and Nutrition Survey (CHNS), which utilizes a multistage, random cluster sampling strategy to collect longitudinal data across 228 communities within 9 provinces of China. A detailed description of the survey design and procedures are available in Zhang B et al. [21]. We accessed eight waves of CHNS surveys conducted between 1991 and 2011, with the final sample comprising 73,110 observations after excluding observations with missing data.

### Measures/variables

The main objective of this study is to compare the impact of individual factors and regional factors on the inequality of health care utilization. The dependent variable measured whether a resident utilized outpatient or inpatient services during the past 4 weeks, and the two key independent variables were the individual’s personal income quintile and the region of residence. Sampled individuals were divided into five income groups, according to their income quintile (top to bottom), and sampled communities were divided into five regional categories, comprising urban, suburban, town, rich village and poor village. Rich versus poor villages were categorized by their per capita income. Following Andersen’s behavioral model of health care utilization, comprising predisposing characteristics (such as demographics, and position within the social structure), enabling characteristics (such as economic status), and need based characteristics (perception of need for health services) [22], we controlled for age, gender, marital status, education level, health insurance and body mass index (BMI). Although self-report health status (SRH) is a widely used proxy variable for health needs [23–25], SRH did not appear in all of the CHNS questionnaires. Since BMI is associated with SRH [26], health-related quality of life [27, 28] and mortality risk [29], BMI was used to proxy health status. In addition, wave dummies for each of 8 collection points between 1991 and 2011 were added into the model to capture the period effects. Table 1 presents definitions for all variables in the analysis.

**Table 1:** Variable definitions

Variable	Variable definitions
Wave	
1991	Reference group
1993	1 if survey conducted at 1993; 0 otherwise
1997	1 if survey conducted at 1997; 0 otherwise
2000	1 if survey conducted at 2000; 0 otherwise
2004	1 if survey conducted at 2004; 0 otherwise
2006	1 if survey conducted at 2006; 0 otherwise
2009	1 if survey conducted at 2009; 0 otherwise
2011	1 if survey conducted at 2011; 0 otherwise
Region group	
Urban	Reference group
Suburban	1 if respondents lived in suburban; 0 otherwise
Town	1 if respondents lived in town; 0 otherwise
Rich village	1 if respondents lived in rich village; 0 otherwise
Poor village	1 if respondents lived in poor village; 0 otherwise
Income group	
Quintile1	Reference group
Quintile2	1 if in the second highest quintile; 0 otherwise
Quintile3	1 if income in the middle quintile; 0 otherwise
Quintile4	1 if income in the second lowest quintile; 0 otherwise
Quintile5	1 if income in the bottom quintile; 0 otherwise
Age group	
0~	Reference group
16~	1 if aged 16–30; 0 otherwise
31~	1 if aged 31–45; 0 otherwise
46~	1 if aged 46–60; 0 otherwise
61~	1 if older than 60; 0 otherwise
Gender	
Male	Reference group
Female	1 if female; 0 otherwise
Marriage status	
Never married	Reference group
Married	1 if married; 0 otherwise
Others	1 if divorced, widowed, separated, or unknow; 0 otherwise
Education level	
Below primary school	Reference group
Grad from primary	1 if grad from primary; 0 otherwise
Junior middle school	1 if grad from junior middle school; 0 otherwise
Senior middle school or above	1 if grad from senior middle school or above; 0 otherwise
Health insurance	
Yes	Reference group
No	1 if no insurance; 0 otherwise
BMI	
Underweight	Reference group
Normal weight	1 if 18.5 < = BMI < 26; 0 otherwise
Overweight	1 if 26 < = BMI < 31; 0 otherwise
Obese	1 if BMI > = 31; 0 otherwise

### Statistical analysis

Data analysis was performed by using the STATA 14.0 (College Station, Texas USA) and carried out by descriptive statistics and the random-effects logit model.

Descriptive statistics for utilizing outpatient and inpatient service were reported as counts and proportions, with corresponding chi-square and the *p*-values, to examine whether there were statistically significant differences between subgroups. Second, we adopted the random effects logit model using panel data to investigate regional disparities. Panel data models can offset potential problems associated with unobserved heterogeneity that may induce inconsistent estimators in cross-sectional models. To get consistent and efficient estimators, panel data analysis involves both fixed effects models and the random-effects models. Some variables in our models do not vary over time, such as gender, occupation, and regional groups, which would be omitted in fixed effects models. Since these variables are important factors explaining healthcare utilization, random-effects models were employed to retain those variables in our model.

The model was specified as:

1 $$ \ln \left(\frac{P_{it}}{1-{P}_{it}}\right)={\beta}_0+{\beta}_1{RG}_{it}+{\beta}_2{IG}_{it}+{\sum \limits}_{k=1}^K{\alpha}_k{x}_{kit}+{\mu}_i $$

where *P*_*it*_ represented the probability of utilization of outpatient and inpatient service of individual *i* at period *t*; *RG*_*it*_ indicated region group; *IG*_*it*_ represented the income group; *β*_0_ was the intercept; coefficients *β*_1_ and *β*_2_ represented region disparities and income disparities. Further, *x*_*kit*_ were control variables, such as age, gender, marital status, education level and so on, where *α*_*k*_ is *k* th regression coefficient; *μ*_*i*_ was the random effect representing the effect of the *i* individual. In addition to exploring the dynamic trend of regional disparities from 1991 to 2011, an interaction term between regional group dummies and wave dummies was added into the model.

## Results

Table 2 displays the descriptive statistics for the variables in the entire sample as well as the outpatient and inpatient samples. During the previous 4 weeks, the probability of using outpatient service was 8.6% and using inpatient services was 1.1%. Table 2 shows that over the period 1991–2011, the probability of using outpatient services and inpatient service utilization first declined and then increased. The probability of using outpatient and inpatient services were significantly (*p* < 0.001) different across regional groups. Urban residents had the highest outpatient and inpatient service utilization, while individuals who lived in rich villages had the lowest probability. The results in Table 2 show that the rate of clinic visits was significantly (*p* < 0.001) different across income groups. The bottom-income group was more likely to use the outpatient service, while the middle-income group was less likely to be outpatients. But, the rates of hospitalization did not vary significantly by income groups. The results also indicated that outpatient and inpatient service utilization were significantly different across all the control variables, except gender.

**Table 2:** Descriptive statistics

Characteristics	Sample	Outpatient		Inpatient	
	N (%)^a^	N (%)^b^	*P*	N (%)^b^	*P*
Wave			< 0.001		< 0.001
1991	9976(13.7)	656(6.6)		158(1.6)	
1993	9139(12.5)	272(3.0)		78(0.9)	
1997	9304(12.7)	427(4.6)		61(0.7)	
2000	9226(12.6)	464(5.0)		43(0.5)	
2004	8023(11.0)	964(12.0)		78(1.0)	
2006	7908(10.8)	897(11.3)		74(0.9)	
2009	8396(11.5)	1011(12.0)		106(1.3)	
2011	11,138(15.2)	1602(14.4)		186(1.7)	
Region group			< 0.001		< 0.001
Urban	11,777(16.1)	1331(11.3)		195(1.7)	
Suburban	12,919(17.7)	1082(8.4)		142(1.1)	
Town	11,734(16.1)	941(8.0)		155(1.3)	
Rich village	18,225(24.9)	1376(7.6)		134(0.7)	
Poor village	18,455(25.2)	1563(8.5)		158(0.9)	
Income group			0.002		0.111
Quintile1	14,620(20.0)	1268(8.7)		176(1.2)	
Quintile2	14,622(20.0)	1210(8.3)		167(1.1)	
Quintile3	14,616(20.0)	1204(8.2)		163(1.1)	
Quintile4	14,629(20.0)	1237(8.5)		142(1.0)	
Quintile5	14,623(20.0)	1374(9.4)		136(0.9)	
Age group			< 0.001		< 0.001
0~	7015(9.6)	210(3.0)		27(0.4)	
16~	13,303(18.2)	510(3.8)		62(0.5)	
31~	20,597(28.2)	1229(6.1)		168(0.8)	
46~	19,374(26.5)	2108(10.9)		207(1.1)	
60~	12,821(17.5)	2236(17.4)		320(2.5)	
Gender			< 0.001		0.741
Male	35,212(48.2)	2704(7.7)		373(1.1)	
Female	37,898(51.8)	3589(9.5)		411(1.1)	
Marriage status			< 0.001		< 0.001
Never married	14,697(20.1)	477(3.3)		62(0.4)	
Married	53,401(73.0)	5037(9.4)		615(1.2)	
Others	5012(6.9)	779(15.5)		107(2.1)	
Education level			< 0.001		< 0.001
Below primary school	19,829(27.1)	2185(11.0)		280(1.4)	
Grad from primary	15,950(21.8)	1361(8.5)		162(1.0)	
Junior middle school	21,723(29.7)	1489(6.9)		184(0.9)	
Senior middle school or above	15,608(21.4)	1258(8.1)		158(1.0)	
Health insurance			< 0.001		< 0.001
Yes	39,239(53.7)	2433(6.2)		248(0.6)	
No	33,871(46.3)	3860(11.4)		536(1.6)	
BMI			< 0.001		< 0.001
Underweight	10,385(14.2)	701(6.8)		78(0.8)	
Normal weight	51,797(70.85)	4243(8.2)		547(1.1)	
Overweight	9692(13.26)	1153(11.9)		142(1.5)	
Obese	1236(1.69)	196(15.9)		17(1.4)	
Over all	73,110(100.0)	6293(8.6)		784(1.1)	

^a^Sample distribution among each character

^b^Number and percentage of health care utilization

**Fig. 1:**
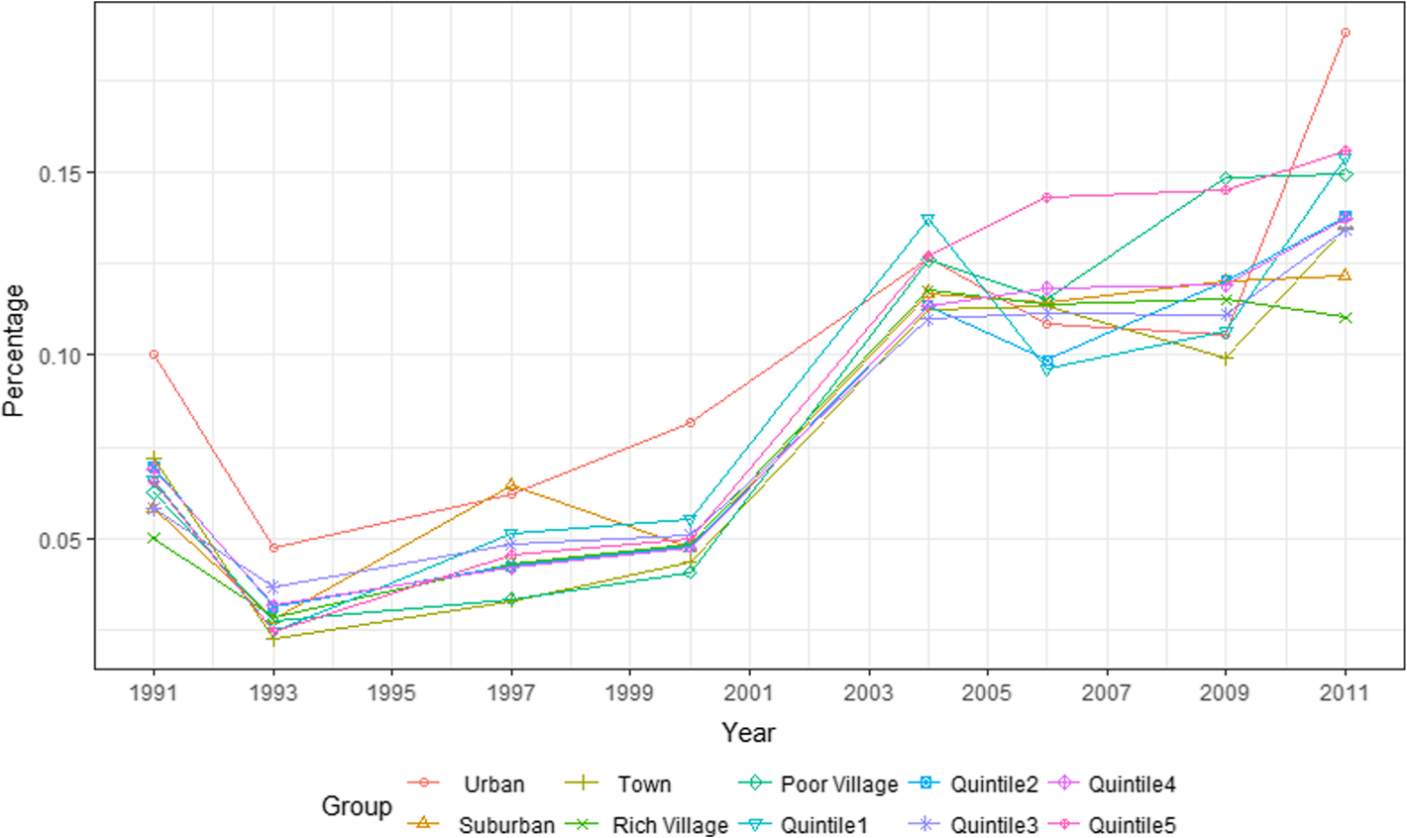
Outpatient service use among income and regional groups in China (1991–2011)

**Fig. 2:**
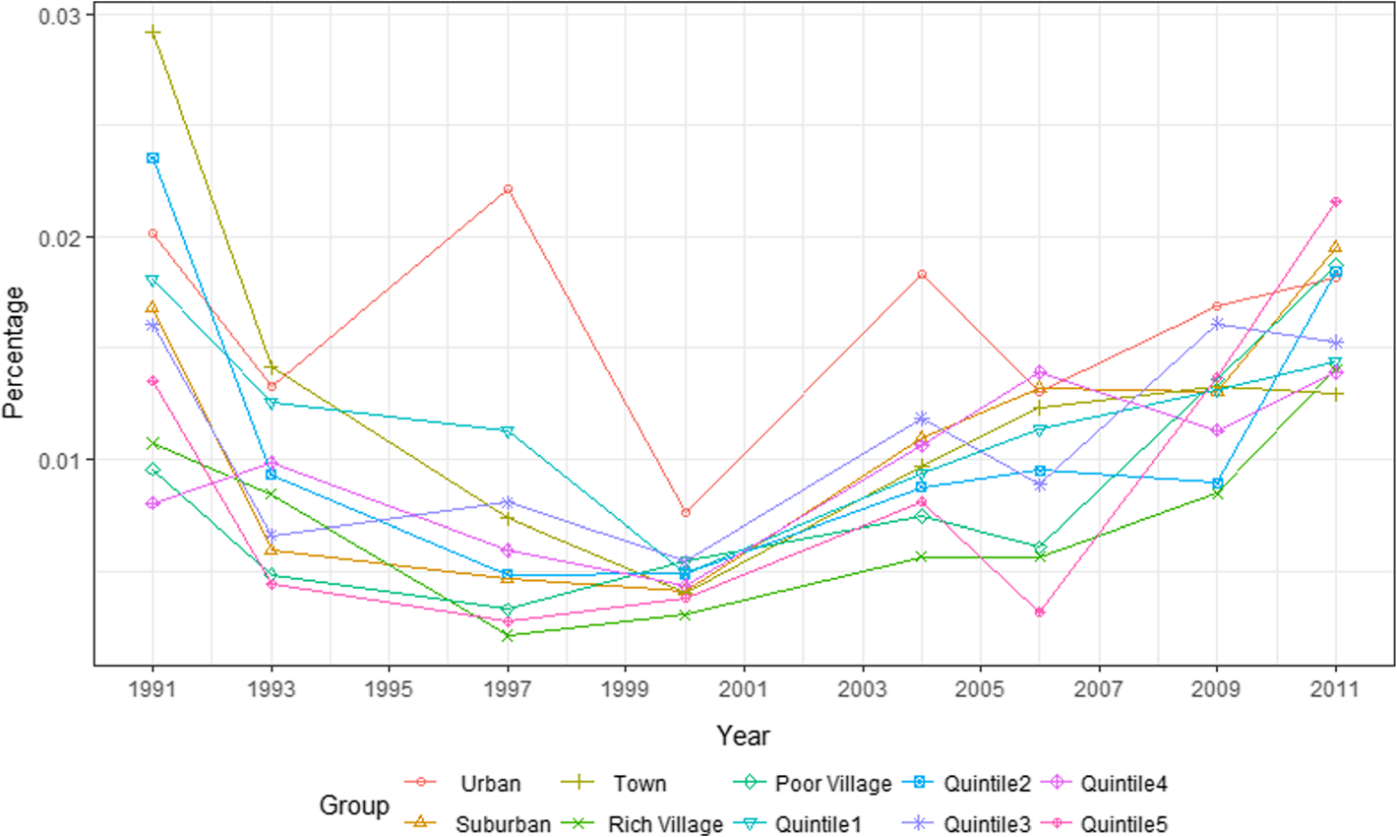
Inpatient service use among income and regional groups in China (1991–2011)

Figures 1 and 2 shown outpatient and inpatient service use among income and regional groups between 1991 and 2011. As shown in the Fig. 1, the disparity in the outpatient rate between income groups was very small before 2004, reaching a minimum in 2000, but increased after 2004. The disparity in the outpatient rate between regional groups decreased before 2006, then began to widen. The two figures demonstrate that the disparity of inpatient use was larger than outpatient use, and the disparity between regional groups was larger than between income groups in most years.

Results of the random effects regressions for income and regional disparities in health care utilization are presented in Table 3, with region and income groups highlighted. As showed in the first column, after controlling for confounding variables, suburban (odds ratio (OR) =0.802, 95% confidence interval (CI): 0.720–0.893), town (OR: 0.722, 95% CI: 0.648–0.804), rich (OR: 0.728, 95% CI: 0.656–0.807) and poor village (OR: 0.778, 95% CI: 0.698–0.868) residents were less likely to use outpatient services, but the differences between income groups were not significant, except the differences between top and bottom income group in outpatient service use (OR: 1.134, 95% CI: 1.021–1.258). The results also show that outpatient service utilization was more likely to occur for those in old age, female and married groups, and was less likely to occur for those with high education levels and from the high BMI group. For inpatient use (column 3 in Table 3), significant differences were observed for rich (OR: 0.609, 95% CI: 0.472–0.785) and poor village residents (OR: 0.752, 95% CI: 0. 576–0.983), but there was no significant difference between income groups. Older age and health insurance increased the probability of hospitalization. When we excluded regional groups in model 2 (columns 2 and 4), the results remained roughly the same. Our results show that the inequalities in healthcare utilization were fundamentally caused by regional, not personal income, disparities.

**Table 3:** Regression results of random effects logit^a^

	Outpatient		Inpatient	
	Model 1	Model 2	Model 1	Model 2
Intercept	0.032 (0.004)^***^	0.025(0.003)^***^	0.004(0.001)^**^	0.003(0.001)^***^
Region group				
Urban	Reference		Reference	
Suburban	0.802(0.044)^***^		0.899(0.112)	
Town	0.722(0.040)^***^		0.939(0.113)	
Rich village	0.728(0.038)^***^		0.609(0.079)^***^	
Poor village	0.778(0.043)^***^		0.752(0.103)^**^	
Income group				
Quintile1	Reference	Reference	Reference	Reference
Quintile2	1.003(0.048)	0.985(0.047)	1.069(0.123)	1.052(0.121)
Quintile3	1.035(0.051)	0.995(0.049)	1.213(0.146)	1.158(0.137)
Quintile4	1.064(0.055)	1.022(0.051)	1.118(0.145)	1.056(0.133)
Quintile5	1.134(0.060)^**^	1.091(0.055)^*^	1.072(0.148)	1.017(0.132)
Age group				
0~	Reference	Reference	Reference	Reference
16~	1.065(0.115)	1.050(0.113)	1.098(0.311)	1.066(0.302)
31~	1.280(0.154)^**^	1.278(0.154)^**^	1.721(0.544)^*^	1.704(0.539)^*^
46~	2.149(0.259)^***^	2.183(0.262)^***^	2.156(0.682)^**^	2.181(0.690)^**^
60~	3.431(0.416)^***^	3.580(0.433)^***^	4.704(1.482)^***^	5.014(1.578)^***^
Gender				
Male	Reference	Reference	Reference	Reference
Female	1.171(0.040)^***^	1.185(0.040)^***^	0.992(0.080)	1.011(0.082)
Marriage status				
Never married	Reference	Reference	Reference	Reference
Married	1.453(0.118)^***^	1.430(0.116)^***^	1.255(0.269)	1.239(0.266)
Others	1.480(0.143)^***^	1.471(0.142)^***^	1.445(0.354)	1.451(0.356)
Education level				
Below primary school	Reference	Reference	Reference	Reference
Grad from primary	0.924(0.042)^*^	0.932(0.042)	0.982(0.109)	0.994(0.110)
Junior middle school	0.775(0.038)^***^	0.801(0.038)^***^	0.865(0.102)	0.914(0.107)
Senior middle school or above	0.767(0.043)^***^	0.835(0.044)^***^	0.795(0.106)^*^	0.913(0.117)
Health insurance	1.273(0.051)^***^	1.299(0.052)^***^	2.213(0.224)^***^	2.336(0.234)^***^
BMI				
Underweight	Reference	Reference	Reference	Reference
Normal weight	0.710(0.040)^***^	0.711(0.040)^***^	0.985(0.142)	1.001(0.144)
Overweight	0.835(0.056)^***^	0.839(0.056)^***^	1.127(0.188)	1.169(0.196)
Obese	1.067(0.116)	1.064(0.115)	0.988(0.291)	1.029(0.303)

^***^ *p* < 0.01; ^**^ *p* < 0.05; ^*^ *p* < 0.1

^a^Cells represent odds ratio (standard error). Categorical variable wave indicating year of survey was included in the model, not shown in the table

**Fig. 3:**
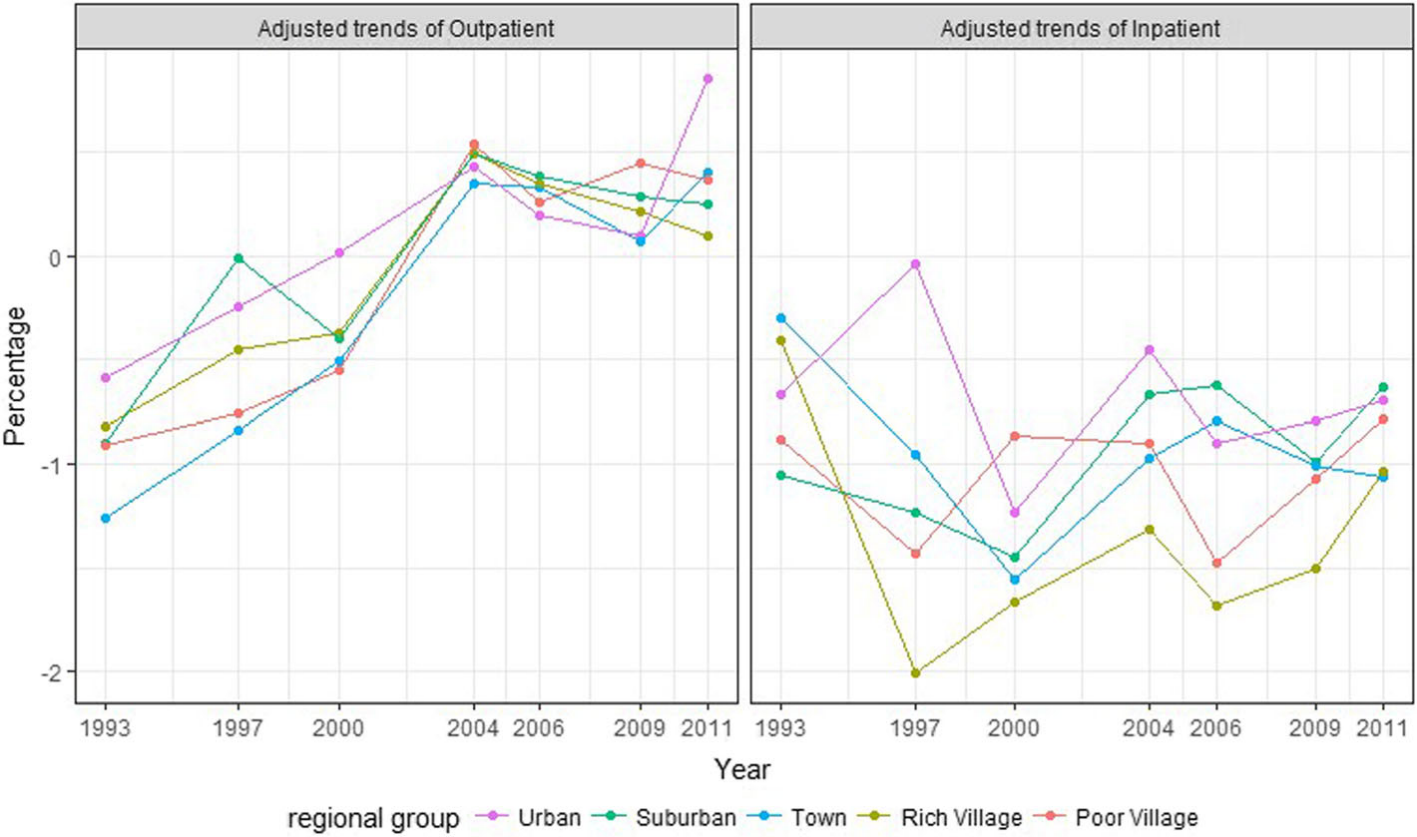
Adjusted trends of health care utilization by regional groups, 1991–2011

Table 4 and Fig. 3 present the dynamic trends of regional disparities in health utilization from 1991 to 2011. The rate of outpatient utilization increased before 2004, and then declined. In urban areas, the rate of increase was much greater than other regions before 2004, and the decline more rapidly from 2004 to 2009. The rate of inpatient health care utilization presented a fluctuating decreasing trend. The rate of decrease was much less in the urban region and much greater in rich villages.

**Table 4:** Adjusted trends in health care utilization by regional groups, 1991-2011^a^

Region	ref.	1993	1997	2000	2004	2006	2009	2011
Clinic Visit								
Urban	0	−0.587(0.151)^***^	−0.244(0.129)^*^	0.014(0.120)	0.425(0.107)^***^	0.195(0.116)^*^	0.097(0.115)	0.847(0.080)^***^
Suburban	0	−0.900(0.166)^***^	−0.012(0.113)	−0.397(0.128)^***^	0.490(0.099)^***^	0.384(0.101)^***^	0.288(0.101)^***^	0.244(0.097)^**^
Town	0	−1.259(0.190)^***^	−0.840(0.165)^***^	−0.503(0.142)^***^	0.347(0.111)^***^	0.327(0.108)^***^	0.067(0.113)	0.400(0.092)^***^
Rich village	0	−0.822(0.132)^***^	−0.455(0.114)^***^	−0.373(0.111)^***^	0.492(0.090)^***^	0.344(0.091)^***^	0.211(0.091)^**^	0.092(0.090)
Poor village	0	−0.906(0.134)^***^	−0.759(0.125)^***^	−0.548(0.118)^***^	0.533(0.088)^***^	0.256(0.091)^***^	0.449(0.087)^***^	0.361(0.086)^***^
Inpatient								
Urban	0	−0.669(0.277)^**^	−0.042(0.216)	−1.229(0.339)^***^	−0.450(0.243)^*^	−0.898(0.289)^***^	−0.795(0.254)^***^	−0.698(0.189)^***^
Suburban	0	−1.055(0.351)^***^	−1.237(0.370)^***^	−1.447(0.394)^***^	−0.666(0.268)^**^	−0.621(0.251)^**^	−0.991(0.254)^***^	−0.633(0.210)^***^
Town	0	−0.301(0.249)	−0.953(0.339)^***^	−1.557(0.426)^***^	−0.971(0.315)^***^	−0.791(0.279)^***^	−1.012(0.268)^***^	−1.062(0.235)^***^
Rich village	0	−0.410(0.241)^*^	−2.006(0.459)^***^	−1.666(0.392)^***^	−1.315(0.321)^***^	−1.678(0.323)^***^	−1.505(0.263)^***^	−1.040(0.211)^***^
Poor village	0	−0.885(0.310)^***^	−1.429(0.370)^***^	−0.864(0.299)^***^	−0.898(0.283)^***^	−1.475(0.311)^***^	−1.069(0.224)^***^	−0.784(0.198)^***^

^a^Cells represent coefficient (standard error)

^*^ *p* < 0.1; ^**^ *p* < 0.05; ^***^ *p* < 0.01

## Discussion

Compared to urban residents, we found suburban, town, rich and poor village residents were less likely to use outpatient services; and rich and poor village residents were less likely to use inpatient health care. Differences between income groups were not significant, except the differences between top and bottom income group in outpatient service use. In the random effects logit models with wave-region interactions, we found that China was making progress in increasing health care resource allocation and improving accessibility. Although the gap between urban and rural regions was closing, the disparity among regions remained significant.

Largely consistent with existing studies, the major determinants of inequality of health care utilization in our study were age, gender, BMI, education, marriage status and health insurance, where the last three were not need-related factors.. Elwell-Sutton found these non-need related factors made the largest pro-rich contributions to health case use [30]. However, our results show that income was not as prominent a factor in health care inequality as in previous studies [4, 30]. While health status proxies health need, and self-report health status (SRH) is a widely used proxy variable for health need [23–25], SRH did not appeared in all of the CHNS questionnaires. CHNS provided consistent data on BMI, and BMI is associated with SRH [26], health-related quality of life [19, 31–34] and mortality risk [29]. Used as a proxy variable of health status, we found BMI had a significant association with outpatient utilization.

Regional factors had a more important impact on health care utilization than individual income. That inequality between socioeconomic regions is more pronounced than between individuals is also true in other countries [19, 31–34]. Van Doorslaer et al. found that location of residence contributed to the inequality of health service utilization in Europe and the US [35]. Brezzi et al. also showed that in addition to individual factors, the characteristics of the region where people live, such as the average skill endowment or employment rate, had a significant impact on the probability of unmet medical needs in selected OECD countries [33]. Devaux illustrated that the utilization of cancer screening services largely depended on the availability of national public screening programs, which varied by region across selected OECD countries [10]. In common with Chinese regional health inequalities [36], regional inequality also occurs in national development [37], economic growth [38], income levels [39] and education [40].

Health care accessibility in remote rural regions lags behind urban regions for several reasons. First, practicing (assistant) physicians per thousand population in rural countries (1.51) were less than half of those in urban cities (3.54) in 2014 [1]. A similar pattern can also be observed in the urban–rural allocation of nurses. Second, geographical factors also contribute to the availability of health care. Zhang et al. found that residents whose houses’ were more than 5 km from the nearest health facilities were less likely to utilize health care services than those whose house was less than 5 km from health facilities [4]. Third, government financial support is highly dependent on the local economy. The central government only contributes a limited share of the financial inputs into health care facilities, so rich provinces invest more in health than poorer provinces. Finally, the uneven quality and accessibility to social resources impacts on health care quality.Social resources including education, public transport and commerce, are vital factors that attract human resource and funding [41]. Health care disparity is one of the demonstrated consequences of socioeconomic inequality [36].

Two turning points were identified in our trend analysis. From 1993 to 1997, disparity of inpatient health care utilizations among regions dramatically increased, with only urban areas displaying an increasing utilization trend, while other regions, especially villages, decreased rapidly (see Fig. 3). In China’s health care reform history, commercialization of public health care was encouraged by the 14^th^ Central Committee of the Chinese Communist Party held in 1992 [42], including profit making, diversification of services and cost recovery. As shown in our results, the commercialization side effects were apparent with increasing inequality in access when health care facilities closed down or were sold to private individuals in rural areas. Medical costs rapidly increased and co-operative medicine collapsed. The second turning point was 2006, where the gap among regions started to close. The main contributor of this trend was the establishment of the New Cooperative Medical Scheme (NCMS). In 2003, NCMS was implemented in rural areas, with about half of the rural counties having roughly a 80% NCMS enrollment rate by June 2006 [43] and 97.5% enrolment rate by 2011 [44]. Pre-NCMS, about 80% of the rural residents was not covered by any form of health insurance [43]. As the results of our study suggest, NCMS significantly improved health care access in rural areas.

Besides demand-side subsidies, policy makers should pay more attention to the equity of health care resource allocation. Governments have mainly focused on explicit pro-poor health policies to correct the inequality of health care by enhancing the affordability of access to health care, such as targeted health sector subsidies for the poor [45] and community-based health insurance [46]. Although health insurance is a key factor in promoting health facility access, our study showed that the gap between high and low-income individuals was nearly closed and income factors lost their significance after adjusting for other impact factors. There are some existing supply-side schemes in China. The most important one is governmental financial reimbursement of capital construction and equipment purchase. However, barriers imped their implementation. First, a variety of pro-poor demand-side subsidies co-exist within four ministries (Health, Social Security, Civil Affairs and Finance), but only two ministries (Health and Finance) are responsible for supply-side subsidies. Second, the amounts of financial inputs into supply-side schemes are usually highly related to local government revenue, which means differential health care spending between rich and poor local governments will see health care gaps between regions perpetuated.

Our findings emphasize supply-side inequality in health care utilization. While income-related inequalities contributed to access to health care facilities, the importance of regional disparities in health care access has been underestimated. We recommend monitoring supply-side factors in health policies. Based on our findings, more hospitals, clinics, physicians and nurses should be allocated to remote rural areas to tackle health care facility availability. If availability is the key bar to health care access, then additional funding to enhance affordability will not significantly improve health care access. China’s 1992 health care reforms that ‘marketized’ hospitals provides a lesson that reminds us how availability affects the health care utilization, regardless of the affordability.

This study has the following limitations. Relying on secondary data, some measures of the dimensions of access to health care, such as affordability, and some potential confounding factors, are missing. Second, all data were based on self-reporting, which might lead to recall and information bias. Thirdly, these data are collected before 2012. External validity is nuanced since health care reform strategies are evolving and new regulations have been launched during the last several years. Lastly, as restricted by secondary data, health care utilization based on need and demand cannot be easily divided, so the results should be interpreted with care.

## Conclusion

We found that regional factors were a more important determinant of inequalities of health care utilization than individual, especially income, factors. Second, availability of services was a more prominent issue in China than affordability. While being cognizant of issues of demand-side subsidies, policy makers should pay increased attention to inequalities in health care utilization arising from resource allocation issues.
